# The genome sequence of the Pinion-streaked Snout, *Schrankia costaestrigalis* (Stephens, 1834)

**DOI:** 10.12688/wellcomeopenres.19402.1

**Published:** 2023-05-10

**Authors:** Douglas Boyes, Peter W.H. Holland, University of Oxford and Wytham Woods Genome Acquisition Lab, Darwin Tree of Life Barcoding collective, Wellcome Sanger Institute Tree of Life programme, Wellcome Sanger Institute Scientific Operations: DNA Pipelines collective, Tree of Life Core Informatics collective, Darwin Tree of Life Consortium

**Affiliations:** 1 UK Centre for Ecology & Hydrology, Wallingford, England, UK; 2 University of Oxford, Oxford, England, UK

**Keywords:** Schrankia costaestrigalis, Pinion-streaked Snout, genome sequence, chromosomal, Lepidoptera

## Abstract

We present a genome assembly from an individual male *Schrankia costaestrigalis* (the Pinion-streaked Snout; Arthropoda; Insecta; Lepidoptera; Erebidae). The genome sequence is 572.0 megabases in span. Most of the assembly is scaffolded into 31 chromosomal pseudomolecules, including the Z sex chromosome. The mitochondrial genome has also been assembled and is 16.1 kilobases in length. Gene annotation of this assembly on Ensembl identified 19,453 protein coding genes.

## Species taxonomy

Eukaryota; Metazoa; Ecdysozoa; Arthropoda; Hexapoda; Insecta; Pterygota; Neoptera; Endopterygota; Lepidoptera; Glossata; Ditrysia; Noctuoidea; Erebidae; Hypeninae; *Schrankia*; *Schrankia costaestrigalis* (Stephens, 1834) (NCBI:txid411963).

## Background

The Pinion-streaked Snout *Schrankia costaestrigalis* is a slender moth in the family Erebidae, found widely across Europe. The species has also been recorded in Russia, China, Japan, the Canary Islands, Australia and New Zealand ( GBIF Secretariat, 2022). *S. costaestrigalis* rests with its narrow forewings held flat over the hindwings giving the moth a triangular appearance; the moth also has prominent palps that project forward. The forewings are pale grey-brown crossed by a darker jagged band outlined in black; this wing pattern is similar to that of a closely related species, the white-line snout *S. taenialis*. A third moth – the ‘Autumnal Snout’ – was originally given species status ( *S. intermedialis*) but later suggested to be a possible naturally-occurring hybrid between *S. costaestrigalis* and *S. taenialis ( Waring *et al.*, 2017)*; support for the hybrid hypothesis comes from a pattern of shared bands produced by PCR amplification of microsatellite DNA ( Anderson *et al.*, 2007). There is scope for testing further the hybrid hypothesis using a larger number of samples and additional genomic markers.

In Britain, *S. costaestrigalis* is found predominantly in marshes, bogs, fens and damp woodland across England, Wales, Scotland and Northern Ireland ( NBN Atlas Partnership, 2022; Thompson & Nelson, 2003). The adult moth is on the wing from June to October in southern England; this extended flight period is thought to reflect two generations per year with the larvae of the second generation overwintering ( Waring *et al*., 2017). There has long been uncertainty over the larval food plant used in the wild. In captivity, larvae have been reared on the flowers of wild thyme *Thymus* sp. ( Waring *et al.*, 2017), with an unverified report that this is supplemented by cannibalistic tendencies ( Stokoe, 1948). More recent findings from China and Japan suggest that the natural food is more likely to be underground roots and tubers. For example, *S. costaestrigalis* has emerged as a new crop pest of potato *Solanum tuberosum* in Guangxi Province, China, with the larvae living underground and eating tubers; at one affected site crop losses approached 90% across almost 300 hectares of crop ( Xian *et al.*, 2022; Zeng *et al.*, 2020). Similarly, in Tanegashima Island, Japan, *S. costaestrigalis* has been recorded as a pest of broad bean *Vicia faba*, again with larvae living underground and eating roots and root nodules ( Yoshimatsu & Nishioka, 1995). The latter authors note that the adult moths can also live in underground spaces in the soil, a habit comparable to the cave-dwelling lifestyle of two *Schrankia* species in Hawaii ( Howarth *et al.*, 2020) and *S. costaestrigalis* in Tenerife ( Oromí, 2018).

We report here the complete genome sequence from a specimen of *S. costaestrigalis* obtained from a single individual collected from a fenland habitat in southern England, scaffolded to chromosome level using chromatin conformation data from a second individual collected at the same site. A genome sequence for this species will facilitate research into the poorly understood biology of this unusual moth and may prove beneficial in designing control strategies when appropriate.

## Genome sequence report

**Figure 1.**
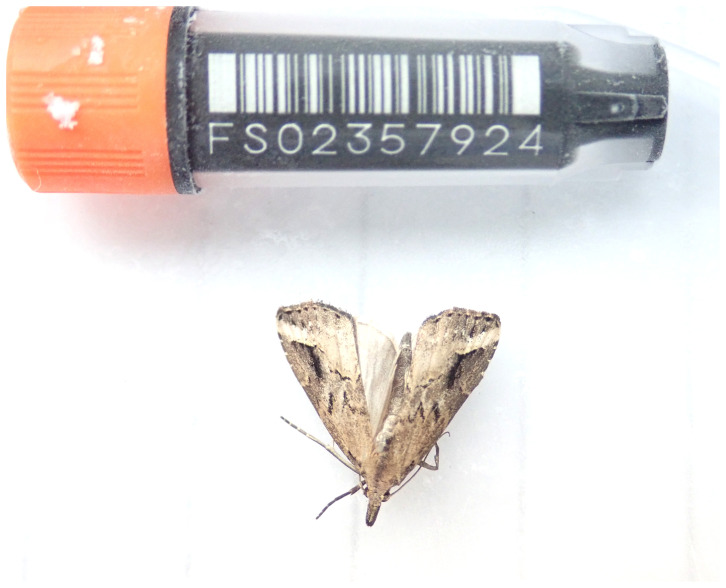
Photograph of the *Schrankia costaestrigalis* (ilSchCost1) specimen used for genome sequencing.

The genome was sequenced from one male *Schrankia costaestrigalis* ( Figure 1) collected from Wytham Woods, Oxfordshire, UK (latitude 51.77, longitude –1.31). A total of 38-fold coverage in Pacific Biosciences single-molecule HiFi long reads and 79-fold coverage in 10X Genomics read clouds were generated. Primary assembly contigs were scaffolded with chromosome conformation Hi-C data. Manual assembly curation corrected 101 missing or mis-joins and removed 34 haplotypic duplications, reducing the assembly length by 2.33% and the scaffold number by 15.09%, and decreasing the scaffold N50 by 4.6%.

**Figure 2.**
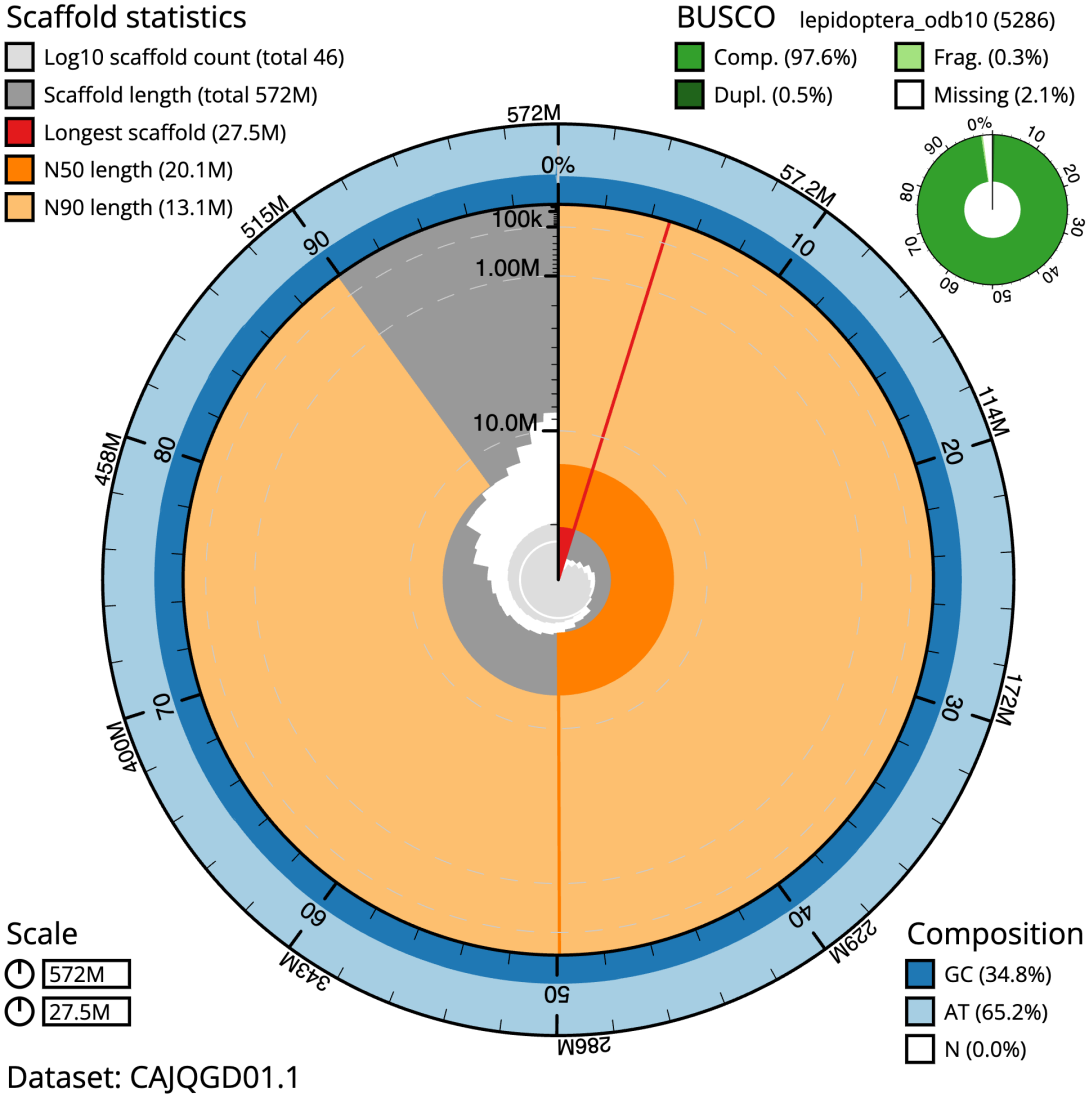
Genome assembly of *Schrankia costaestrigalis*, ilSchCost1.1: metrics. The BlobToolKit Snailplot shows N50 metrics and BUSCO gene completeness. The main plot is divided into 1,000 size-ordered bins around the circumference with each bin representing 0.1% of the 571,991,991 bp assembly. The distribution of scaffold lengths is shown in dark grey with the plot radius scaled to the longest scaffold present in the assembly (27,469,003 bp, shown in red). Orange and pale-orange arcs show the N50 and N90 scaffold lengths (20,082,482 and 13,139,496 bp), respectively. The pale grey spiral shows the cumulative scaffold count on a log scale with white scale lines showing successive orders of magnitude. The blue and pale-blue area around the outside of the plot shows the distribution of GC, AT and N percentages in the same bins as the inner plot. A summary of complete, fragmented, duplicated and missing BUSCO genes in the lepidoptera_odb10 set is shown in the top right. An interactive version of this figure is available at https://blobtoolkit.genomehubs.org/view/ilSchCost1.1/dataset/CAJQGD01.1/snail.

The final assembly has a total length of 572.0 Mb in 45 sequence scaffolds with a scaffold N50 of 20.1 Mb ( Table 1). Most (99.91%) of the assembly sequence was assigned to 31 chromosomal-level scaffolds, representing 30 autosomes and the Z sex chromosome. Chromosome-scale scaffolds confirmed by the Hi-C data are named in order of size ( Figure 2– Figure 5; Table 2). While not fully phased, the assembly deposited is of one haplotype. Contigs corresponding to the second haplotype have also been deposited. The mitochondrial genome was also assembled and can be found as a contig within the multifasta file of the genome submission.

**Table 1.** Genome data for *Schrankia costaestrigalis*, ilSchCost1.1.

Project accession data		
Assembly identifier	ilSchCost1.1	
Species	*Schrankia costaestrigalis*	
Specimen	ilSchCost1	
NCBI taxonomy ID	411963	
BioProject	PRJEB43806	
BioSample ID	SAMEA7520193	
Isolate information	ilSchCost1, male: whole organism (genome sequencing); ilSchCost2, whole organism (Hi-C scaffolding)	
Assembly metrics *		*Benchmark*
Consensus quality (QV)	60.7	*≥ 50*
*k*-mer completeness	100%	*≥ 95%*
BUSCO **	C:97.6%[S:97.1%,D:0.5%], F:0.3%,M:2.1%,n:5,286	*C ≥ 95%*
Percentage of assembly mapped to chromosomes	99.91%	*≥ 95%*
Sex chromosomes	Z chromosome	*localised homologous pairs*
Organelles	Mitochondrial genome assembled	*complete single alleles*
Raw data accessions		
PacificBiosciences SEQUEL II	ERR6606790	
10X Genomics Illumina	ERR6054657–ERR6054660	
Hi-C Illumina	ERR6054661–ERR6054663	
Genome assembly		
Assembly accession	GCA_905475405.1	
*Accession of alternate haplotype*	GCA_905475335.1	
Span (Mb)	572.0	
Number of contigs	86	
Contig N50 length (Mb)	14.4	
Number of scaffolds	45	
Scaffold N50 length (Mb)	20.1	
Longest scaffold (Mb)	27.5	
Genome annotation		
Number of protein-coding genes	19,453	
Number of gene transcripts	19,668	

* Assembly metric benchmarks are adapted from column VGP-2020 of “Table 1: Proposed standards and metrics for defining genome assembly quality” from ( Rhie *et al.*, 2021). ** BUSCO scores based on the lepidoptera_odb10 BUSCO set using v5.3.2. C = complete [S = single copy, D = duplicated], F = fragmented, M = missing, n = number of orthologues in comparison. A full set of BUSCO scores is available at https://blobtoolkit.genomehubs.org/view/ilSchCost1.1/dataset/CAJQGD01.1/busco.

**Figure 3.**
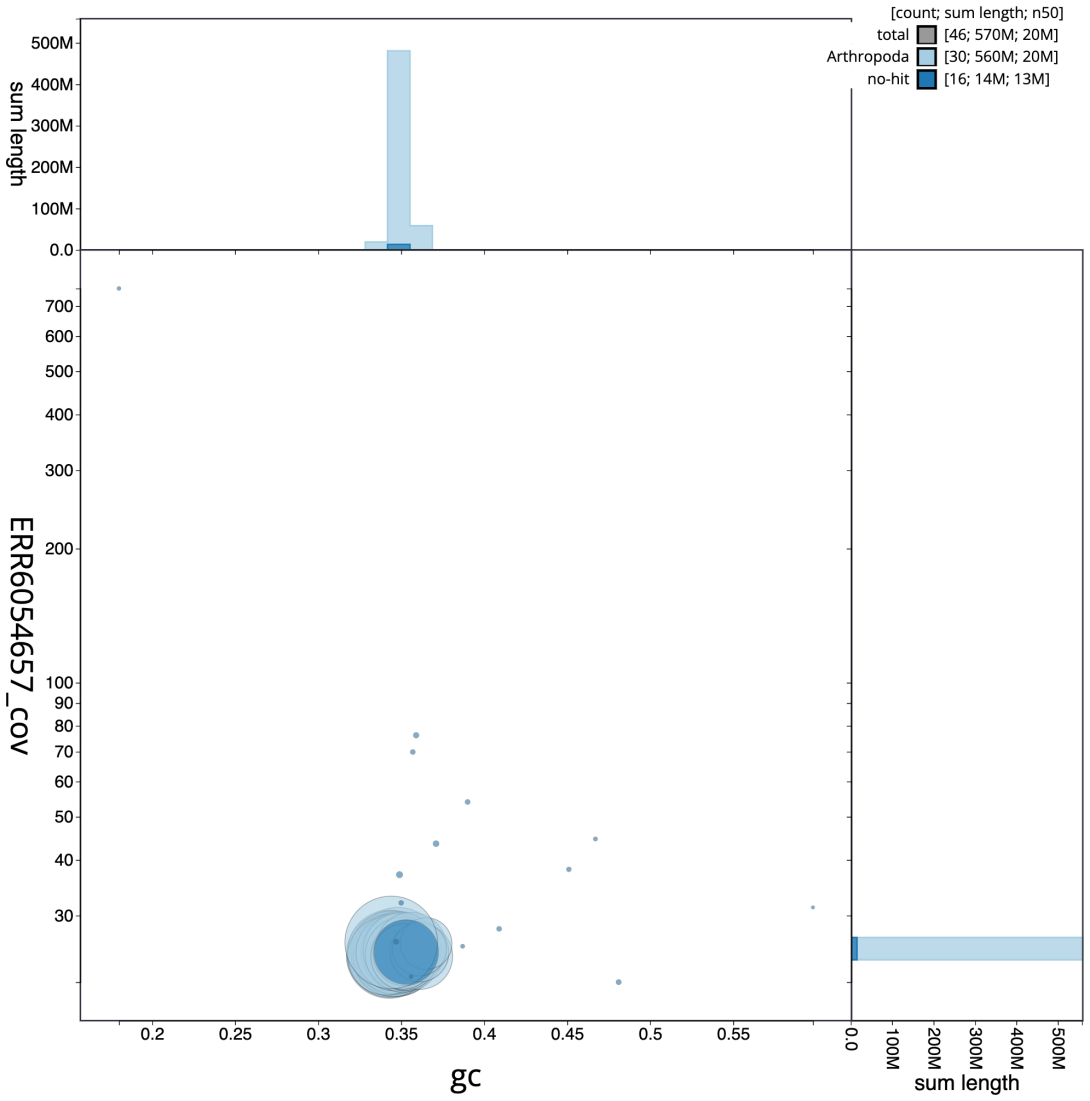
Genome assembly of *Schrankia costaestrigalis*, ilSchCost1.1: GC coverage. BlobToolKit GC-coverage plot. Scaffolds are coloured by phylum. Circles are sized in proportion to scaffold length. Histograms show the distribution of scaffold length sum along each axis. An interactive version of this figure is available at https://blobtoolkit.genomehubs.org/view/ilSchCost1.1/dataset/CAJQGD01.1/blob.

**Figure 4.**
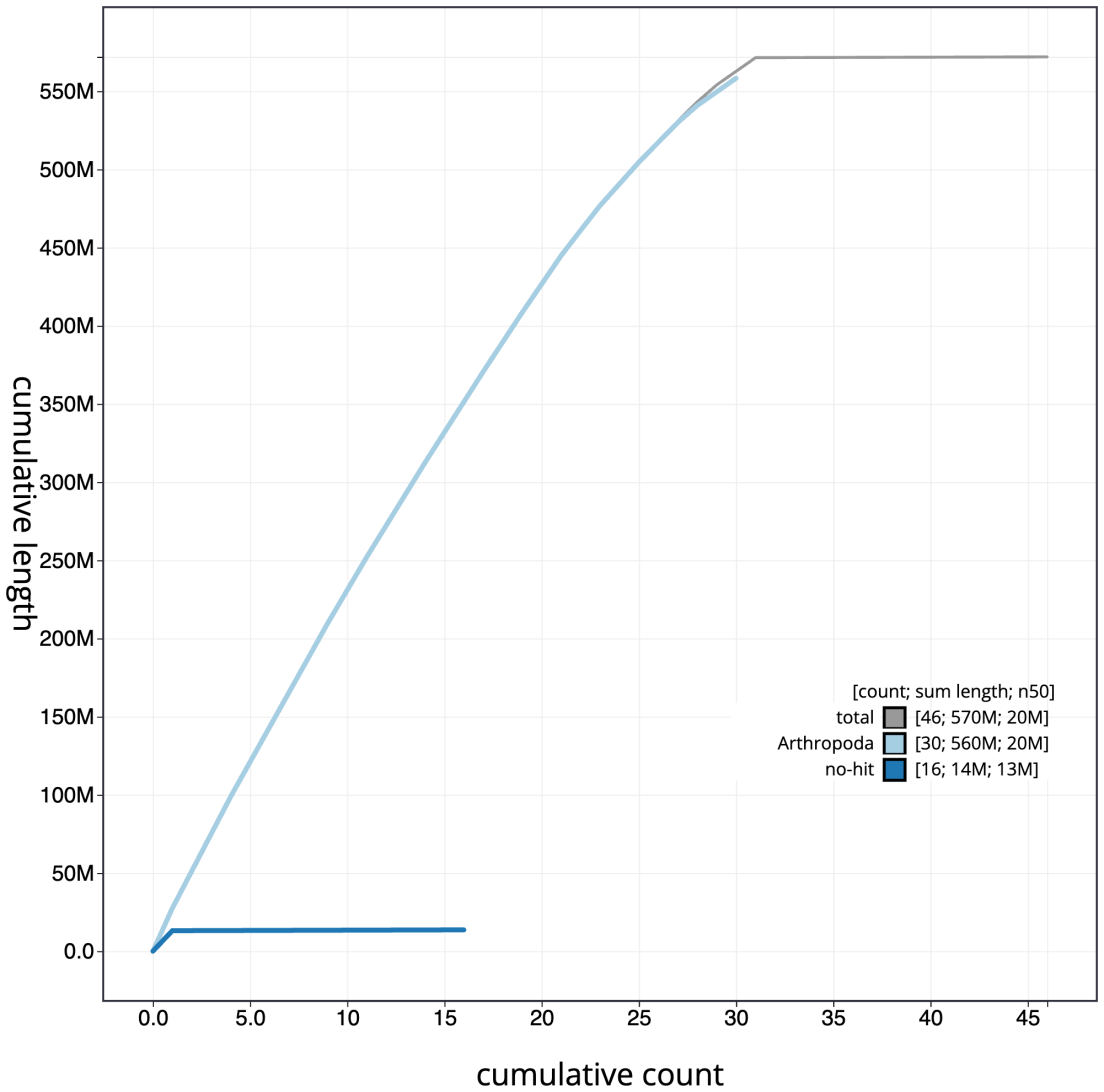
Genome assembly of *Schrankia costaestrigalis*, ilSchCost1.1: cumulative sequence. BlobToolKit cumulative sequence plot. The grey line shows cumulative length for all scaffolds. Coloured lines show cumulative lengths of scaffolds assigned to each phylum using the buscogenes taxrule. An interactive version of this figure is available at https://blobtoolkit.genomehubs.org/view/ilSchCost1.1/dataset/CAJQGD01.1/cumulative.

**Figure 5.**
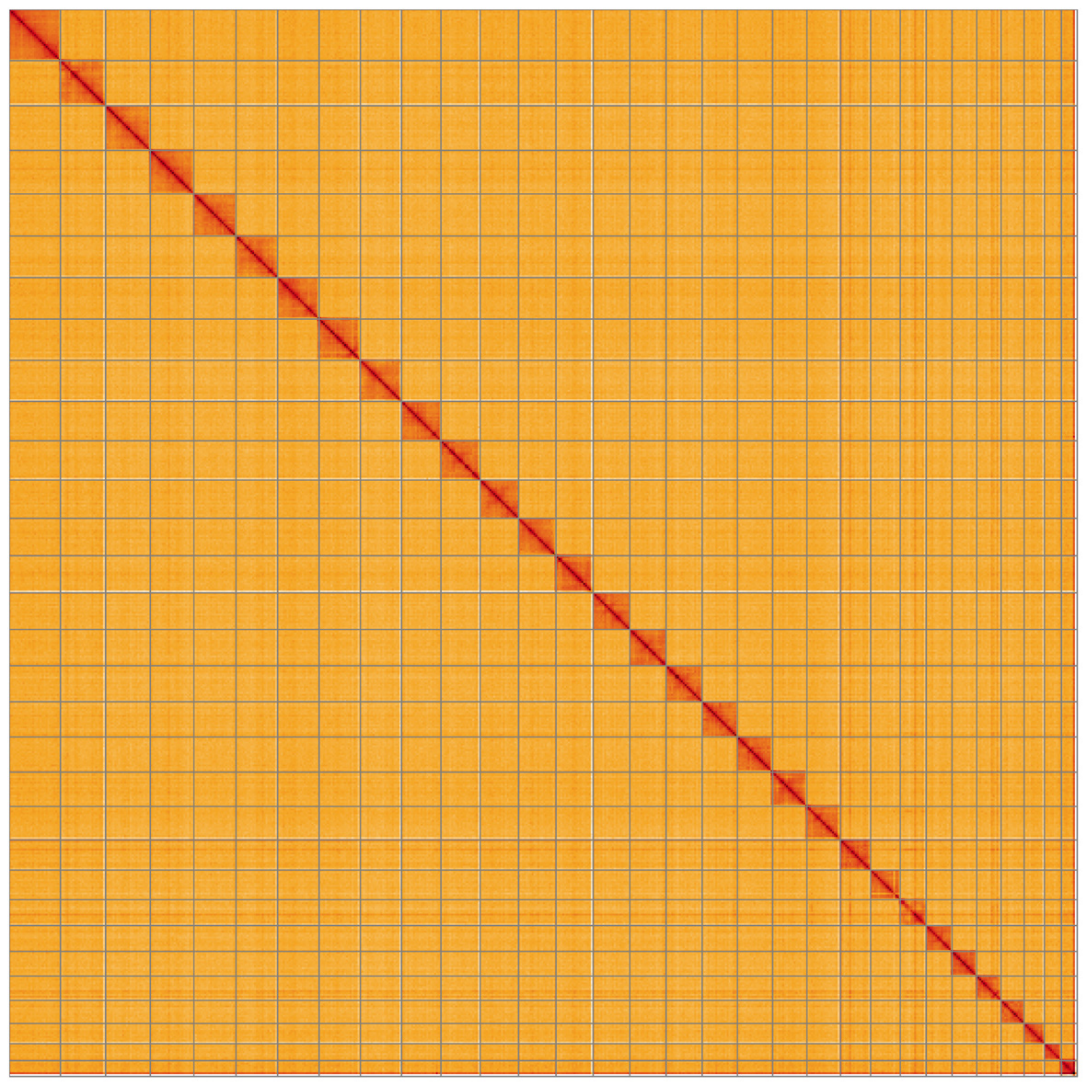
Genome assembly of *Schrankia costaestrigalis*, ilSchCost1.1: Hi-C contact map. Hi-C contact map of the ilSchCost1.1 assembly, visualised using HiGlass. Chromosomes are shown in order of size from left to right and top to bottom. An interactive version of this figure may be viewed at https://genome-note-higlass.tol.sanger.ac.uk/l/?d=NlebYlC5SAKV3EjzWxZAzA.

**Table 2.** Chromosomal pseudomolecules in the genome assembly of *Schrankia costaestrigalis*, ilSchCost1.

INSDC accession	Chromosome	Size (Mb)	GC%
FR997826.1	1	24.12	34.8
FR997827.1	2	23.9	34.3
FR997828.1	3	23.35	34.7
FR997829.1	4	22.57	34.3
FR997830.1	5	22.21	34.5
FR997831.1	6	22.19	34.5
FR997832.1	7	22.19	34.5
FR997833.1	8	21.95	34.3
FR997834.1	9	21.15	34.7
FR997835.1	10	21.01	34.4
FR997836.1	11	20.49	34.5
FR997837.1	12	20.08	34.9
FR997838.1	13	19.87	34.8
FR997839.1	14	19.57	34.5
FR997840.1	15	19.41	34.6
FR997841.1	16	19.31	34.1
FR997842.1	17	18.91	34.9
FR997843.1	18	18.8	34.6
FR997844.1	19	18.41	35
FR997845.1	20	17.94	35.1
FR997846.1	21	16.14	35.6
FR997847.1	22	15.81	35.3
FR997848.1	23	13.93	36.1
FR997849.1	24	13.88	35.2
FR997850.1	25	13.14	35.3
FR997851.1	26	13.07	35.1
FR997852.1	27	12.32	35.2
FR997853.1	28	10.86	36.1
FR997854.1	29	9.08	36.1
FR997855.1	30	8.37	36.5
FR997825.1	Z	27.47	34.4
FR997856.1	MT	0.02	18.7
-	unplaced	0.5	38.9

The estimated Quality Value (QV) of the final assembly is 60.7 with *k*-mer completeness of 100%, and the assembly has a BUSCO v5.3.2 completeness of 97.6% (single = 97.1%, duplicated = 0.5%), using the lepidoptera_odb10 reference set ( *n* = 5,286).

Metadata for specimens, spectral estimates, sequencing runs, contaminants and pre-curation assembly statistics can be found at https://links.tol.sanger.ac.uk/species/411963.

## Genome annotation report

The *Schrankia costaestrigalis* genome assembly GCA_905475405.1 (ilSchCost1.1) as annotated using the Ensembl rapid annotation pipeline ( Table 1; https://rapid.ensembl.org/Schrankia_costaestrigalis_GCA_905475405.1/Info/Index). The resulting annotation includes 19,668 transcribed mRNAs from 19,453 protein-coding genes.

## Methods

### Sample acquisition and nucleic acid extraction

Two *Schrankia costaestrigalis* specimens (ilSchCost1 and ilSchCost2) were collected from Wytham Woods, Oxfordshire (biological vice-county: Berkshire), UK (latitude 51.77, longitude –1.31) on 24 August 2019. The specimens were caught using a light trap in fenland habitat by Douglas Boyes (University of Oxford). The specimens were identified by the collector using a field identification, and then snap-frozen on dry ice. Individual ilSchCost1 (specimen Ox000214) was used for genome sequencing, while ilSchCost2 (specimen Ox000215) was used for Hi-C scaffolding.

DNA was extracted at the Tree of Life laboratory, Wellcome Sanger Institute (WSI). The ilSchCost1 sample was weighed and dissected on dry ice. Whole organism tissue was disrupted using a Nippi Powermasher fitted with a BioMasher pestle. High molecular weight (HMW) DNA was extracted using the Qiagen MagAttract HMW DNA extraction kit. Low molecular weight DNA was removed from a 20 ng aliquot of extracted DNA using the 0.8X AMpure XP purification kit prior to 10X Chromium sequencing; a minimum of 50 ng DNA was submitted for 10X sequencing. HMW DNA was sheared into an average fragment size of 12–20 kb in a Megaruptor 3 system with speed setting 30. Sheared DNA was purified by solid-phase reversible immobilisation using AMPure PB beads with a 1.8X ratio of beads to sample to remove the shorter fragments and concentrate the DNA sample. The concentration of the sheared and purified DNA was assessed using a Nanodrop spectrophotometer and Qubit Fluorometer and Qubit dsDNA High Sensitivity Assay kit. Fragment size distribution was evaluated by running the sample on the FemtoPulse system.

### Sequencing

Pacific Biosciences HiFi circular consensus and 10X Genomics read cloud DNA sequencing libraries were constructed according to the manufacturers’ instructions. DNA sequencing was performed by the Scientific Operations core at the WSI on Pacific Biosciences SEQUEL II (HiFi) and HiSeq X Ten (10X) instruments. Hi-C data were generated from whole organism tissue of ilSchCost2 using the Arima2 kit and sequenced on the HiSeq X Ten instrument.

### Genome assembly, curation and evaluation

Assembly was carried out with Hifiasm ( Cheng *et al.*, 2021) and haplotypic duplication was identified and removed with purge_dups ( Guan *et al.*, 2020). One round of polishing was performed by aligning 10X Genomics read data to the assembly with Long Ranger ALIGN, calling variants with FreeBayes ( Garrison & Marth, 2012). The assembly was then scaffolded with Hi-C data ( Rao *et al.*, 2014) using YaHS ( Zhou *et al.*, 2023) SALSA2 ( Ghurye *et al.*, 2019). The assembly was checked for contamination and corrected using the gEVAL system ( Chow *et al.*, 2016) as described previously ( Howe *et al.*, 2021). Manual curation was performed using gEVAL, HiGlass ( Kerpedjiev *et al.*, 2018) and Pretext ( Harry, 2022). The mitochondrial genome was assembled using MitoHiFi ( Uliano-Silva *et al.*, 2022), which performed annotation using MitoFinder ( Allio *et al.*, 2020). To evaluate the assembly, MerquryFK was used to estimate consensus quality (QV) scores and *k*-mer completeness ( Rhie *et al.*, 2020). The genome was analysed and BUSCO scores ( Manni *et al.*, 2021; Simão *et al.*, 2015) were generated within the BlobToolKit environment ( Challis *et al.*, 2020). Table 3 contains a list of software tool versions and sources.

**Table 3.** Software tools: versions and sources.

Software tool	Version	Source
BlobToolKit	4.0.7	https://github.com/blobtoolkit/blobtoolkit
BUSCO	5.3.2	https://gitlab.com/ezlab/busco
FreeBayes	1.3.1-17- gaa2ace8	https://github.com/freebayes/freebayes
gEVAL	N/A	https://geval.org.uk/
Hifiasm	0.12	https://github.com/chhylp123/hifiasm
HiGlass	1.11.6	https://github.com/higlass/higlass
Long Ranger ALIGN	2.2.2	https://support.10xgenomics.com/genome-exome/software/ pipelines/latest/advanced/other-pipelines
MitoHiFi	2	https://github.com/marcelauliano/MitoHiFi
PretextView	0.2	https://github.com/wtsi-hpag/PretextView
purge_dups	1.2.3	https://github.com/dfguan/purge_dups
SALSA	2.2	https://github.com/salsa-rs/salsa

### Genome annotation

The BRAKER2 pipeline ( Brůna *et al.*, 2021) was used in the default protein mode to generate annotation for the *Schrankia costaestrigalis* assembly (GCA_905475405.1, Apr 2021) in Ensembl Rapid Release.

### Ethics and compliance issues

The materials that have contributed to this genome note have been supplied by a Darwin Tree of Life Partner. The submission of materials by a Darwin Tree of Life Partner is subject to the Darwin Tree of Life Project Sampling Code of Practice. By agreeing with and signing up to the Sampling Code of Practice, the Darwin Tree of Life Partner agrees they will meet the legal and ethical requirements and standards set out within this document in respect of all samples acquired for, and supplied to, the Darwin Tree of Life Project. All efforts are undertaken to minimise the suffering of animals used for sequencing. Each transfer of samples is further undertaken according to a Research Collaboration Agreement or Material Transfer Agreement entered into by the Darwin Tree of Life Partner, Genome Research Limited (operating as the Wellcome Sanger Institute), and in some circumstances other Darwin Tree of Life collaborators.

## Data Availability

European Nucleotide Archive: *Schrankia costaestrigalis* (pinion-streaked snout). Accession number PRJEB43806; https://identifiers.org/ena.embl/PRJEB43806. ( Wellcome Sanger Institute, 2021) The genome sequence is released openly for reuse. The *Schrankia costaestrigalis* genome sequencing initiative is part of the Darwin Tree of Life (DToL) project. All raw sequence data and the assembly have been deposited in INSDC databases. Raw data and assembly accession identifiers are reported in Table 1.

## Author information

Members of the University of Oxford and Wytham Woods Genome Acquisition Lab are listed here: https://doi.org/10.5281/zenodo.4789928.

Members of the Darwin Tree of Life Barcoding collective are listed here: https://doi.org/10.5281/zenodo.4893703.

Members of the Wellcome Sanger Institute Tree of Life programme are listed here: https://doi.org/10.5281/zenodo.4783585.

Members of Wellcome Sanger Institute Scientific Operations: DNA Pipelines collective are listed here: https://doi.org/10.5281/zenodo.4790455.

Members of the Tree of Life Core Informatics collective are listed here: https://doi.org/10.5281/zenodo.5013541.

Members of the Darwin Tree of Life Consortium are listed here: https://doi.org/10.5281/zenodo.4783558.
