# Supplementary material for: The genome sequence of the Pinion-streaked Snout, Schrankia costaestrigalis (Stephens, 1834)
Source: Wellcome Open Res. 2023 May 10;8:203. [Version 1] doi: 10.12688/wellcomeopenres.19402.1 (PMC11109538; doi:10.12688/wellcomeopenres.19402.1)
Supplement: Peer review history

# Reviewer report: Reviewer response for version 1

**DOI:** 10.21956/wellcomeopenres.21491.r80008

**Published:** 2024-11-11

**Authors:** Antonio S Ortiz

**Affiliations:** 1 University of Murcia, Murcia, Spain

Boyes & Holland describes the genome assembly *Schrankia costaestrigalis* (Stephens, 1834) and provides important genomic information about its size (near 572 Mb) in 31 chromosomes. A new genome sequenced from the moth family Erebidae is added to others species of the genus as *Hypena proboscidalis *(Linnaeus, 1758) by Boyes & Holland (2021) published in Wellcome Open Research (WOR). This assembly provides valuable infomation for the genomics of Lepidoptera and for further phylogenetic research in the evolutionary pattern in the family Erebidae. The methods for the sequencing and assembly used are appropriate and technically supported.

Are sufficient details of methods and materials provided to allow replication by others?

Yes

Is the rationale for creating the dataset(s) clearly described?

Yes

Are the datasets clearly presented in a useable and accessible format?

Yes

Are the protocols appropriate and is the work technically sound?

Yes

Reviewer Expertise:

Molecular genetics, systematics and conservation on Lepidoptera

I confirm that I have read this submission and believe that I have an appropriate level of expertise to confirm that it is of an acceptable scientific standard.

# Reviewer report: Reviewer response for version 1

**DOI:** 10.21956/wellcomeopenres.21491.r71883

**Published:** 2024-05-14

**Authors:** Benoit Nabholz

**Affiliations:** 1 Universitaire de France (IUF), Paris, France

The authors present the chromosome scale assembly of the pinion-striped snout,

*Schrankia costaestrigalis*.

 The genome is of high quality and the pipeline is the same as for the other genomes produced by the Darwin Tree of Life Project (DToL). All links and datasets are publicly available.

 Comments:

 - The abstract indicates that the genome assembly is from a single male, whereas the method indicates that two individuals were used.

 - Interestingly, the number of annotated CDS (using the BRAKER2 pipeline in Ensembl Rapid Release) varies significantly between Lepidoptera in the DtoL. Here it is in the higher range (~19000), whereas it is often around (~16000). This suggests that these annotations include proteins associated with the transposable element.

 - Should the link to the annotation available in the Ensembl Rapid Release be included in the Data Availability section?

Are sufficient details of methods and materials provided to allow replication by others?

Yes

Is the rationale for creating the dataset(s) clearly described?

Yes

Are the datasets clearly presented in a useable and accessible format?

Yes

Are the protocols appropriate and is the work technically sound?

Yes

Reviewer Expertise:

Molecular evolution

I confirm that I have read this submission and believe that I have an appropriate level of expertise to confirm that it is of an acceptable scientific standard.

# Reviewer report: Reviewer response for version 1

**DOI:** 10.21956/wellcomeopenres.21491.r80006

**Published:** 2024-05-02

**Authors:** Nicolas Dowdy

**Affiliations:** 1 Milwaukee Public Museum, Milwaukee, Wisconsin, USA

The article provides a good discussion of what is known about moths in the genus Schrankia (S. costaestrigalis in particular) and presents a new genomic resource for this economically important species. The quality of the results are somewhat lower than comparable Erebid genomes produced by this group in their past work, however this genome appears to be of high quality at current standards.

Are sufficient details of methods and materials provided to allow replication by others?

Yes

Is the rationale for creating the dataset(s) clearly described?

Yes

Are the datasets clearly presented in a useable and accessible format?

Yes

Are the protocols appropriate and is the work technically sound?

Yes

Reviewer Expertise:

Evolution and Systematics of Lepidoptera

I confirm that I have read this submission and believe that I have an appropriate level of expertise to confirm that it is of an acceptable scientific standard.
